# Using integrated geophysics data set to delineate Phetchabun active fault in Thailand

**DOI:** 10.1016/j.dib.2020.105608

**Published:** 2020-04-22

**Authors:** Rungroj Arjwech, Wanwisa Boonsungnern, Pakawat Sriwangpon, Kittipong Somchat, Potpreecha Pondthai

**Affiliations:** a Department of Geotechnology, Faculty of Technology, Khon Kaen University, Khon Kaen, 40002, Thailand.; b Department of Geology and Geophysics, Texas A&M University, College Station, Texas, 77843-3115, USA

**Keywords:** 2D-3D electrical resistivity tomography, Gravity survey, PHETCHABUN Fault

## Abstract

In order to precisely locate active faults in Phetchabun basin, measured data started with a remote sensing and digital elevation model (DEM) methods in order to find traces of potentially active faults and potential sites for field investigations. Geomorphological and geological field investigations were subsequently conducted according to remote sensing method and map interpretations in order to identify suitable sites for gravity survey and 2D-3D electrical resistivity tomography. From this dataset, the gravity models reveal subsurface structures of density contrast and can identify vertical fault plane. The ERT inversion images show zones of high and low resistivity interpreted as fault plane. Integrated three geophysical surveys can provide useful information of the active faults, which have implications on paleo-earthquake investigation in Phetchabun basin.

Specifications Table

**Table:** 

Subject	Earth and Planetary Sciences
Specific subject area	Geophysics; 2-3D Electrical resistivity tomography and gravity surveys
Type of data	Figure, Excel sheet, Data (.dat) and Binary (.bin) files
How data were acquired	The gravity data were acquired using Gravity meter Scintrex CG-5 (Autograv Gravity Meter) by Geometric. The ERT data were acquired using Syscal Pro Resistivity Meter with 96 electrodes by IRIS Instruments.
Data format	Raw
Parameters for data collection	Gravity stations spacing was 5 m. For ERT survey, Dipole-Dipole array for 2D and pol-pole array for 3D were utilized with electrode separations and line spacing of 5 m respectively.
Description of data collection	The survey lines were selected according to the available site accessibility across the interpreted fault lines in west-east direction.
Data source location	The site is located at Ban Huana (1), Lomsak District and Ban Sambon (2), Muang Districts, Phetchabun Province. UTM Grid 720000–760000E and 182000–185000N
Data accessibility	With the article (http://dx.doi.org/10.17632/zhwx8nb7gj.1)

## Value of the data

• The methods to get data are cost-effective, rapid, non-destructive and generates relevant, spatially continuous subsurface information.
• The datasets can be used for characterisation of the subsurface structures which is useful to locate the hidden fault.
• The integrated data are extremely applicable in subsurface investigation such as mineral exploration and groundwater.
The data are used to precisely determine an optimal trenching site with implications for paleo-earthquake data in Phetchabun basin.

## 1 Data Description

**Fig. 1:**
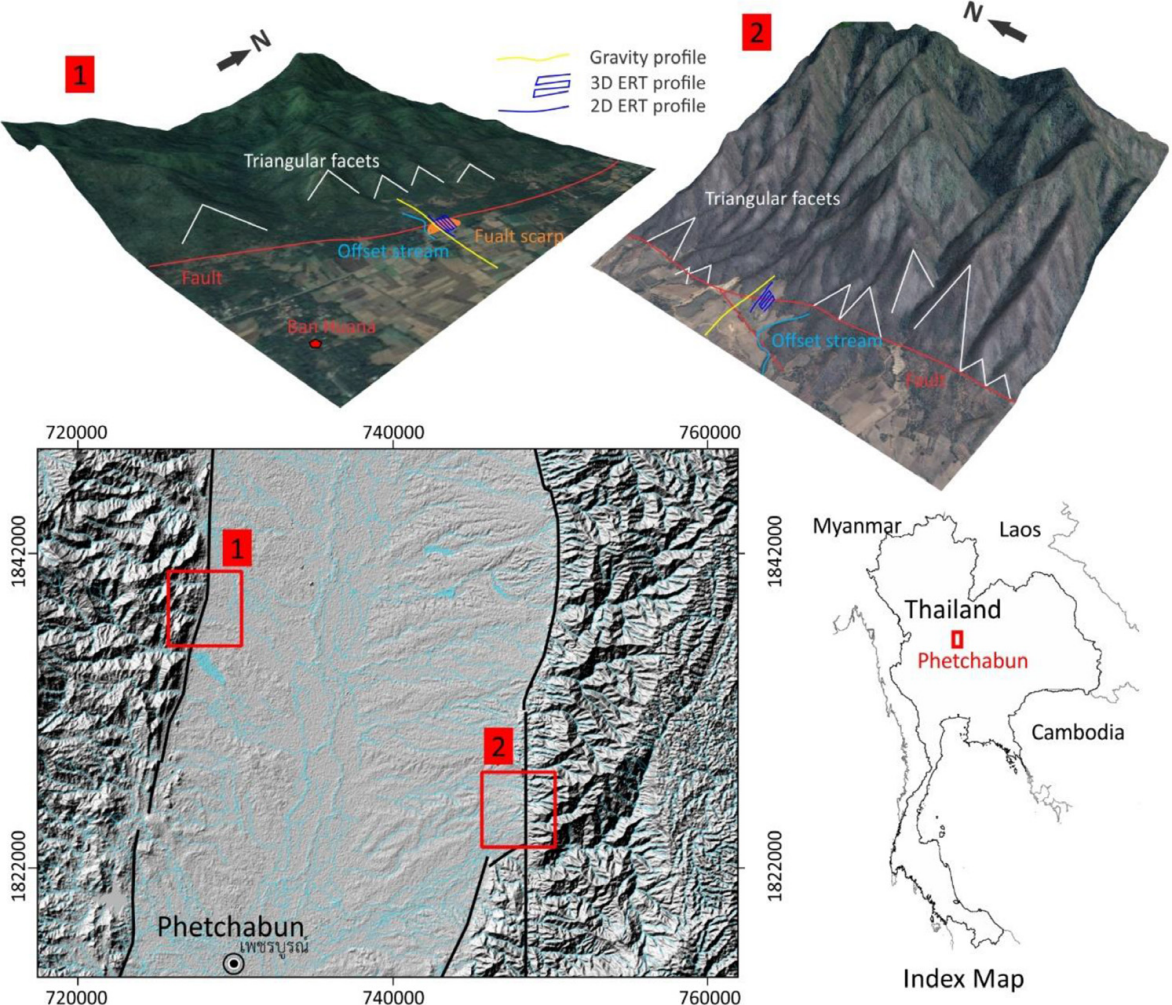
The DEM and 3D map of Phetchabun basin and locations of data measurement in Phetchabun Province, Thailand, a N-S graben as a result of a regional crustal extension along east-west direction [8], [9].

**Fig. 2:**
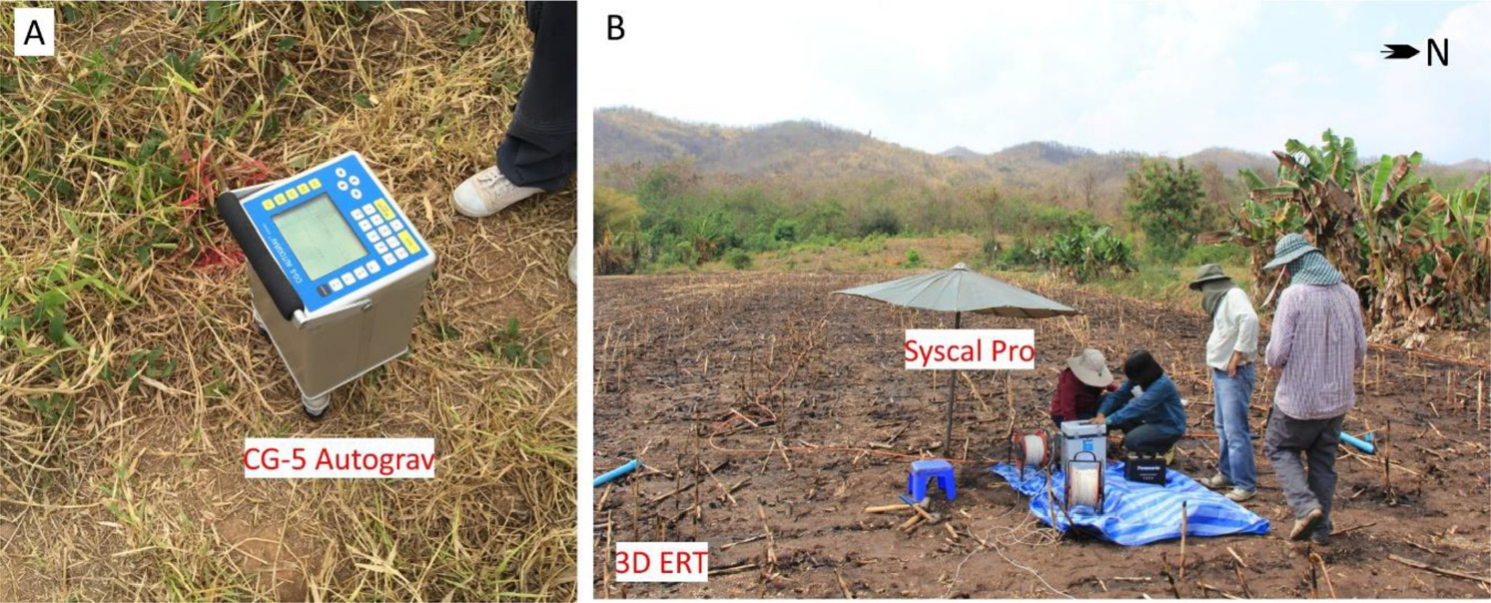
Some representative photographs of the equipment and field deployment, gravity (A) and 3D ERT (B) surveys at the location 1.

**Fig. 3:**
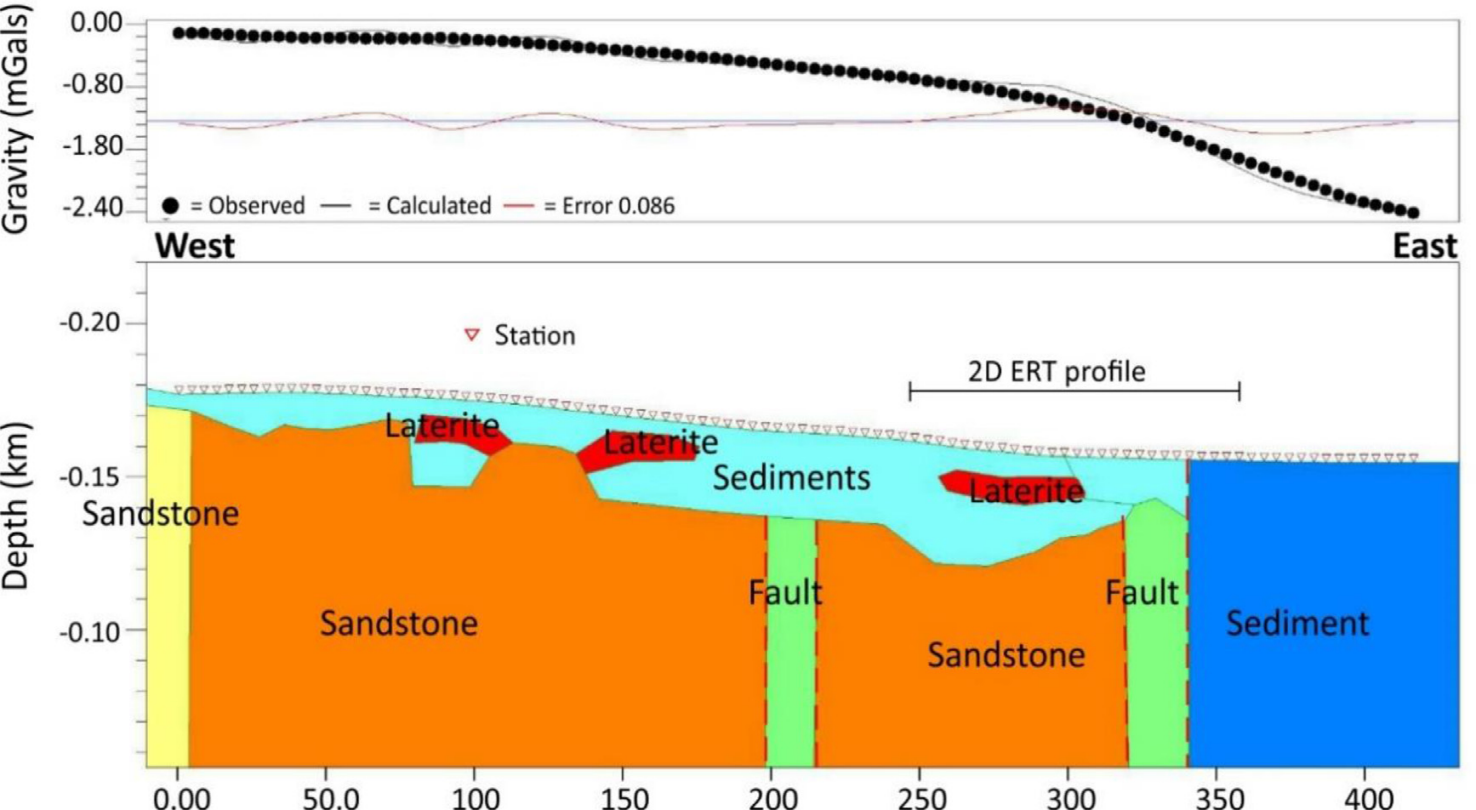
Gravity profile and corresponding model for the profile at location 1.

**Fig. 4:**
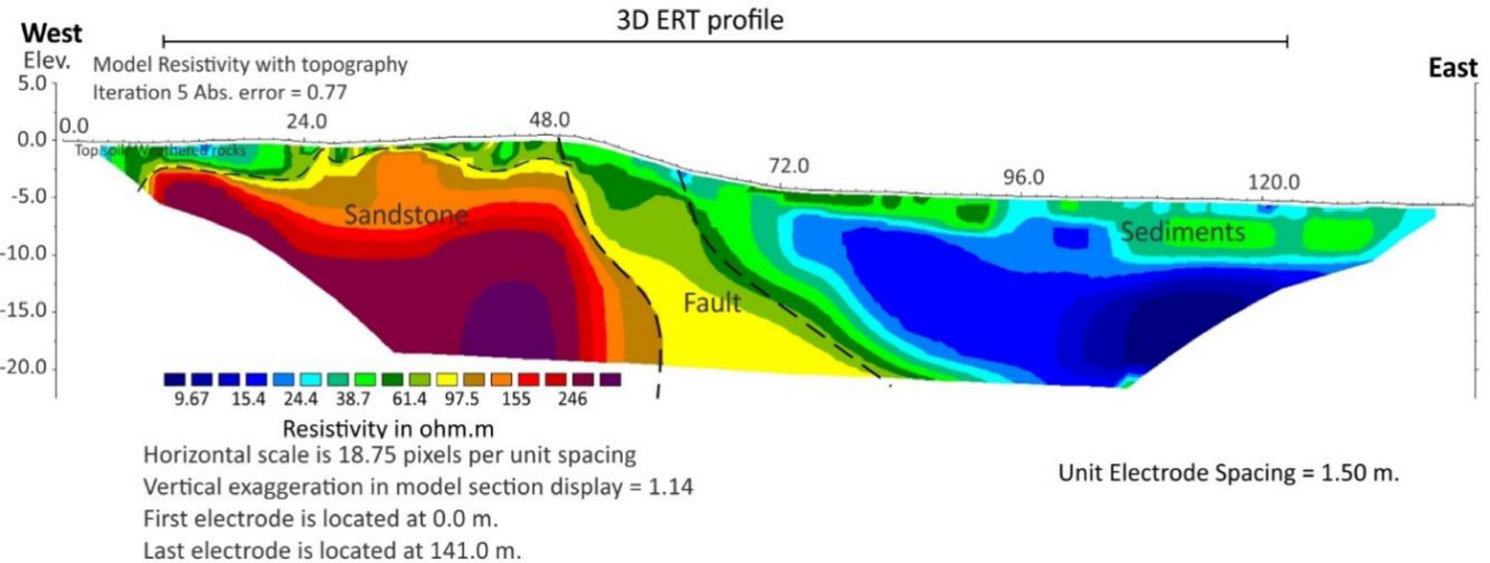
Interpretation of the geoelectrical section of 2D ERT for the profile at location 1.

**Fig. 5:**
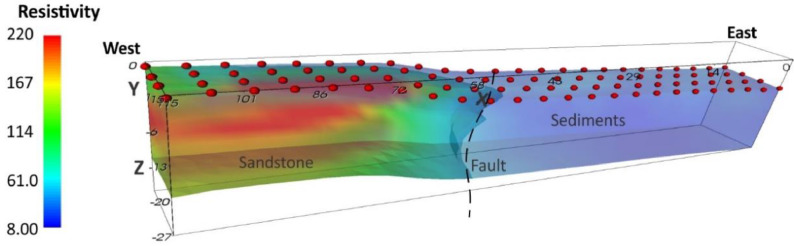
Interpretation of the geoelectrical section of 3D ERT for the profile at location 1.

The gravity and 2D-3D ERT [1], [2], [3], [4], [5], [6] data measurement was carried out across the interpreted fault lines (Fig. 1, Fig. 2). The gravity data were measured and recorded in .raw file. They later were provided in excel sheet mainly consisting of station number, reading time, gravity data, and elevation. The ERT data were recorded in .bin file mainly consisting of apparent resistivity, electric current, potential, and locations of electrodes. Elevations along the survey profiles were measured and added to resistivity data that later were converted to .dat file. Data processing and interpretation were presented as cross section in Fig. 3–5. The attached raw data, files, (Appendices A) consist of gravity and 2D-3D ERT data sets.

## 2 Experimental Design, Materials, and Methods

Phetchabun Fault is identified as active located in Phetchabun basin, Thailand [7] (Fig. 1). Changing on the Earth surface by geological processes can conceal potentially hazardous faults without any indication of their presence at the surface. In order to locate fault locations on both sides of Phetchabun basin, remote sensing and digital elevation model (DEM) methods were carried out to find traces of potentially active faults and potential sites for field investigations. Geomorphological and geological field investigations were subsequently conducted according to the DEM method and map interpretations in order to identify suitable sites for the gravity and 2D-3D electrical resistivity tomography survey methods.

The Microgravity Meter CG-5 Autograv was used. More than 200 gravity reading points along two profiles at location 1 and 2 were carried out perpendicular to the main traces of the fault in west-east profiles (Fig. 2A). The detail needed with separation between stations was 5 m. Elevation of the gravity reading points was measured with a Total Station SANDING ARC6. Latitude and elevation correction were applied for the observed gravity data to obtain free-air gravity. After removing the effects of topographic masses, the data set was reduced to Bouguer gravity anomaly. Gravity models (Fig. 3) were constructed with each model based on a calculated anomaly. Using the Geosoft software, when the calculated and the observed residual anomaly presented the best fit. The corresponding model was considered more realistic.

The Syscal Pro Plus with internal switchbox and an array of 96 steel electrodes was used for ERT survey. The ERT profiles were coincident with the gravity profiles. The 2D ERT data were collected along two profiles. The dipole-dipole array configuration was used with electrode spacing 3 and 5 m and the length is 141 and 235 m respectively. The 3D ERT data were collected along two profiles (Fig. 2B). The pole-pole array configuration was used with electrode and line spacing at 5 m respectively. The survey area was 115 × 15 m. Elevation data were also measured along the resistivity profiles. Res2Dinv and ERTlab64 inversion software were used to read and process 2D and 3D data respectively. They were used for apparent resistivity pseudo-section plotting, data editing and inversion. The inversion algorithm, based on least-square method, used to obtain the subsurface resistivity distribution from the apparent resistivity data. While actual earth structure is always heterogeneous, the 3D image can be reliably interpreted in terms of the principal subsurface geological elements of interest. The ERT inversion images were shown as 2D cross section and 3D volume with interpretation shown in Fig. 4 and 5.

Gravity and detailed ERT data were analysed to provide more constraints on the geometry of the Phetchabun Fault. The depth section, or profile, is purposely designed to be consistent with the measurements and, hence, it can be interpreted in terms of diagnostic soil and rock's bulk density and resistivity. Gravity interpretation involves inversion of data into model. The gravity anomalies can be interpreted from model as function of variations in rocks densities. The resistivity contrast can be discriminated between the presence of sediments with very low resistivity and resistive bedrock with very high resistivity, these are considered in this paper.

The 2D gravity model (Fig. 3) shows that the fault plane is vertical with normal movement related to extensional tectonic event. The 2D and 3D ERT inversion images (Fig. 4 and 5) provide higher resolution images that show the fault plane between high resistivity values of hard rocks and low resistivity values of sediments. These are useful information for further investigation on paleo-earthquake in Phetchabun basin.

## Appendix Supplementary materials

Image, application 1

Image, application 2

Image, application 3
