# Supplementary figures and images for: Using integrated geophysics data set to delineate Phetchabun active fault in Thailand
Source: Data Brief. 2020 Apr 22;30:105608. doi: 10.1016/j.dib.2020.105608 (PMC7200228; doi:10.1016/j.dib.2020.105608)

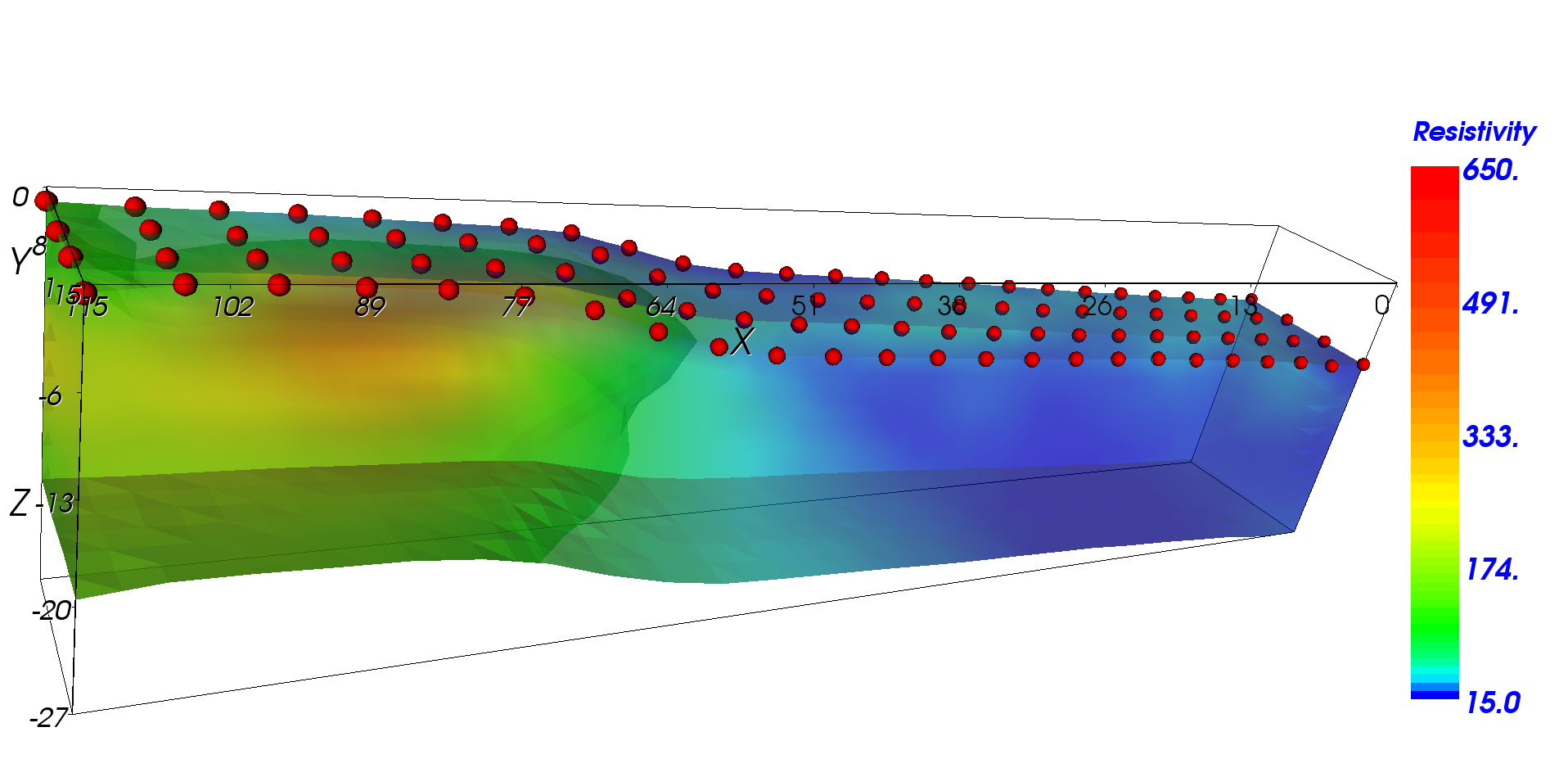

Supplement: Supplementary file 1 [file mmc1.zip › ERT Location 1/3D ERT/Ban-Huana-3D Topo3.ERTprj/Huana.bmp]

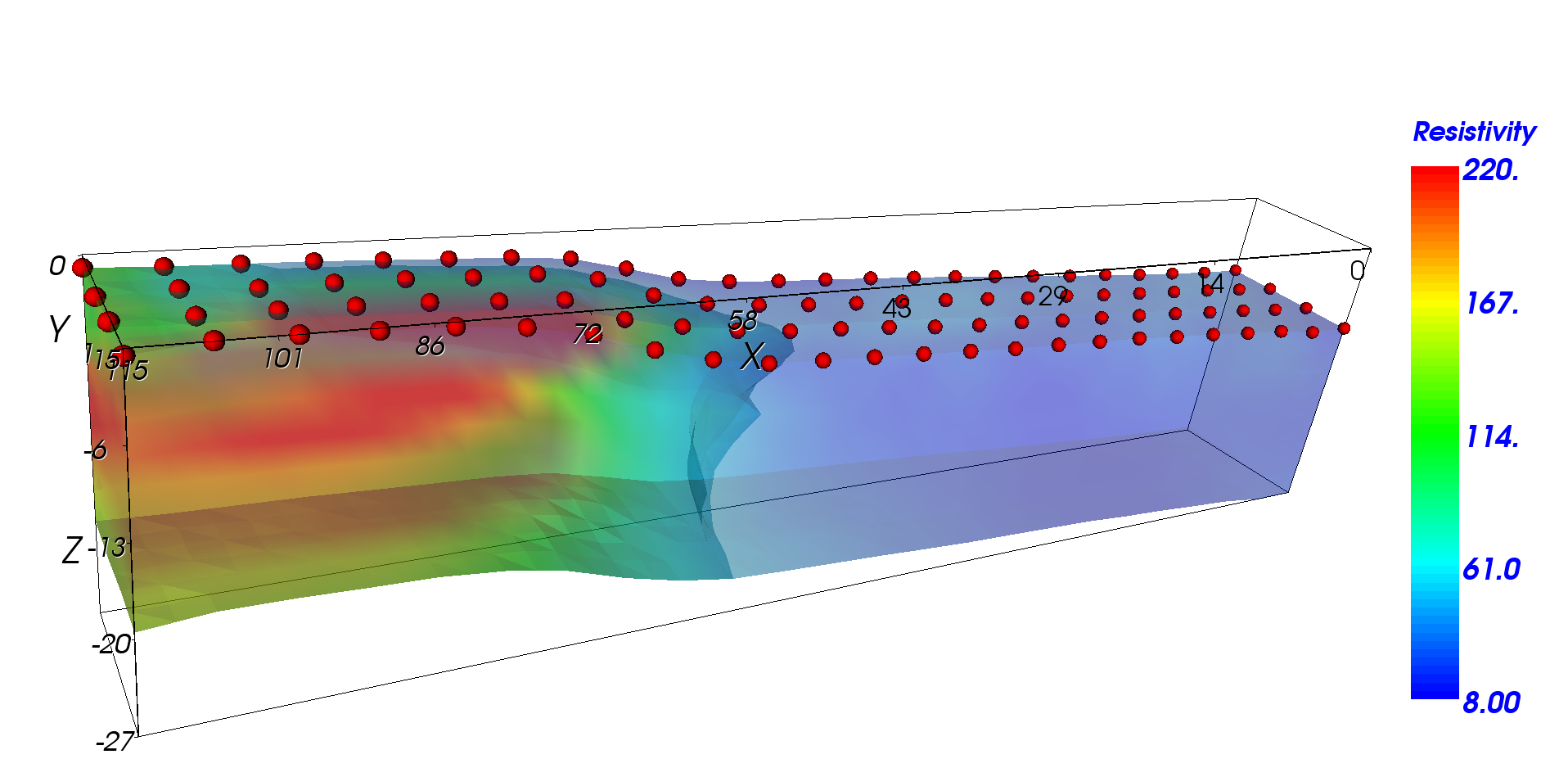

Supplement: Supplementary file 1 [file mmc1.zip › ERT Location 1/3D ERT/Ban-Huana-3D Topo3.ERTprj/Huana1.bmp]

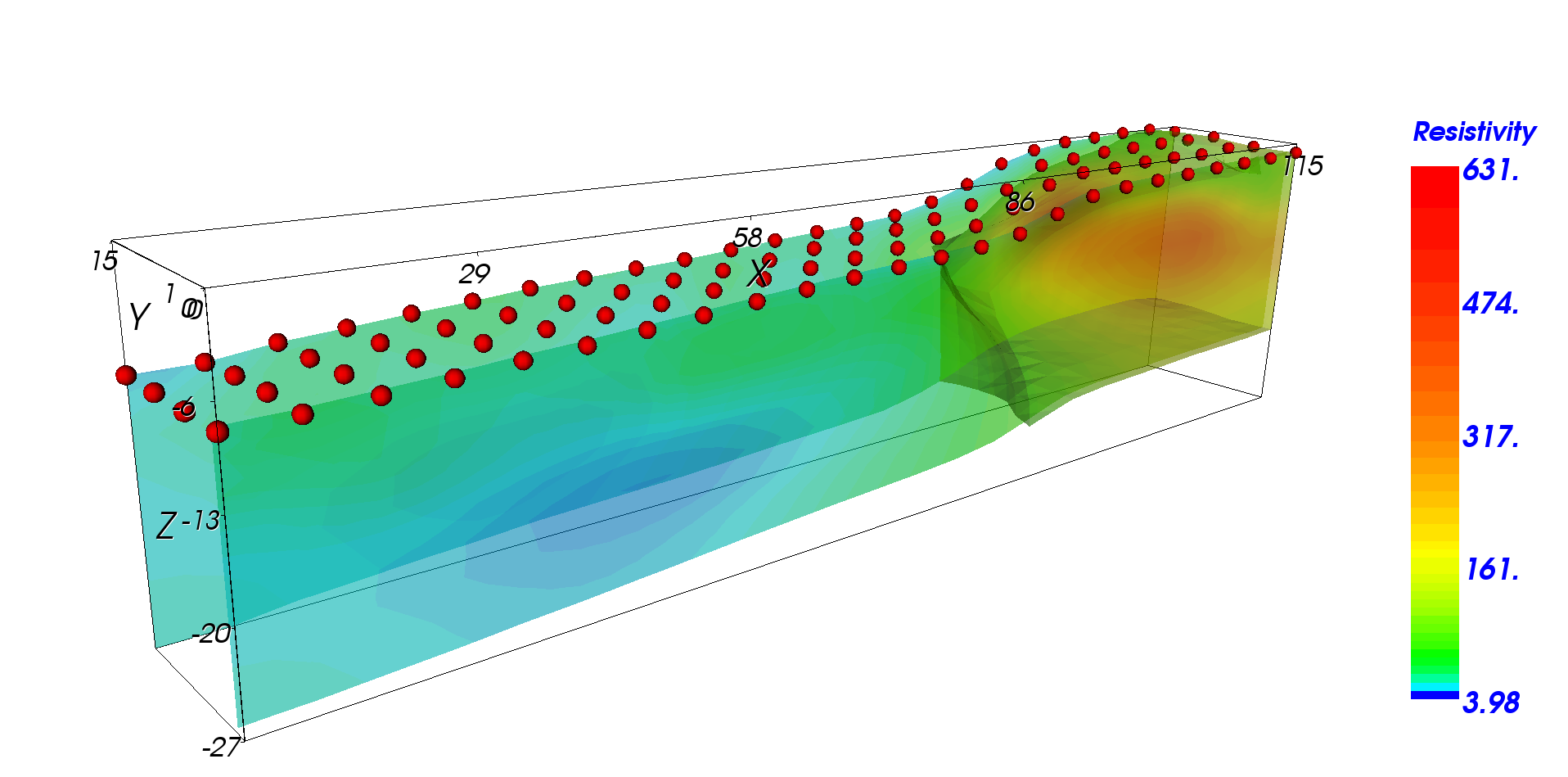

Supplement: Supplementary file 1 [file mmc1.zip › ERT Location 1/3D ERT/Ban-Huana-3D Topo3.ERTprj/Huana2.bmp]

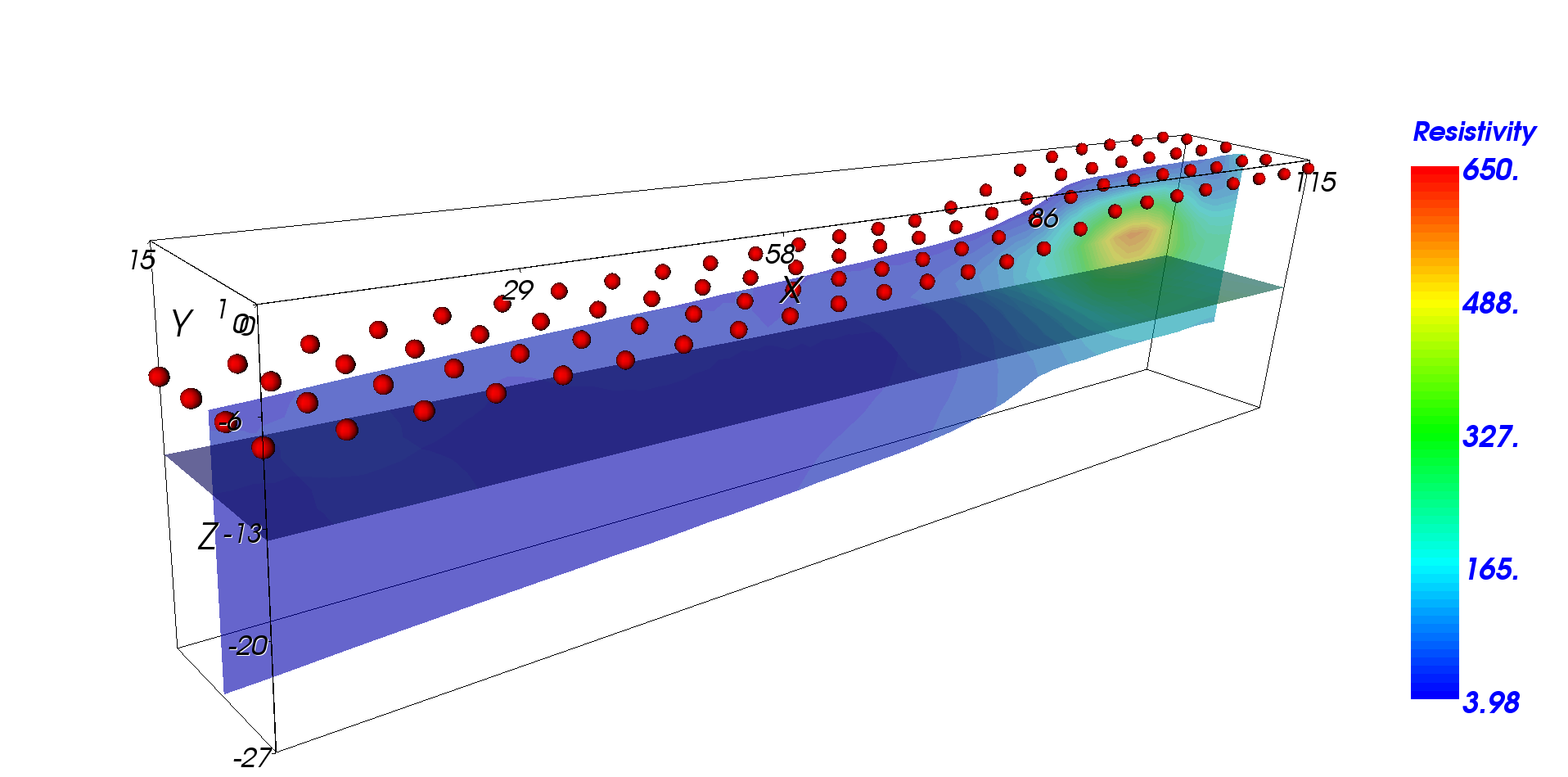

Supplement: Supplementary file 1 [file mmc1.zip › ERT Location 1/3D ERT/Ban-Huana-3D Topo3.ERTprj/Huana3-1.bmp]

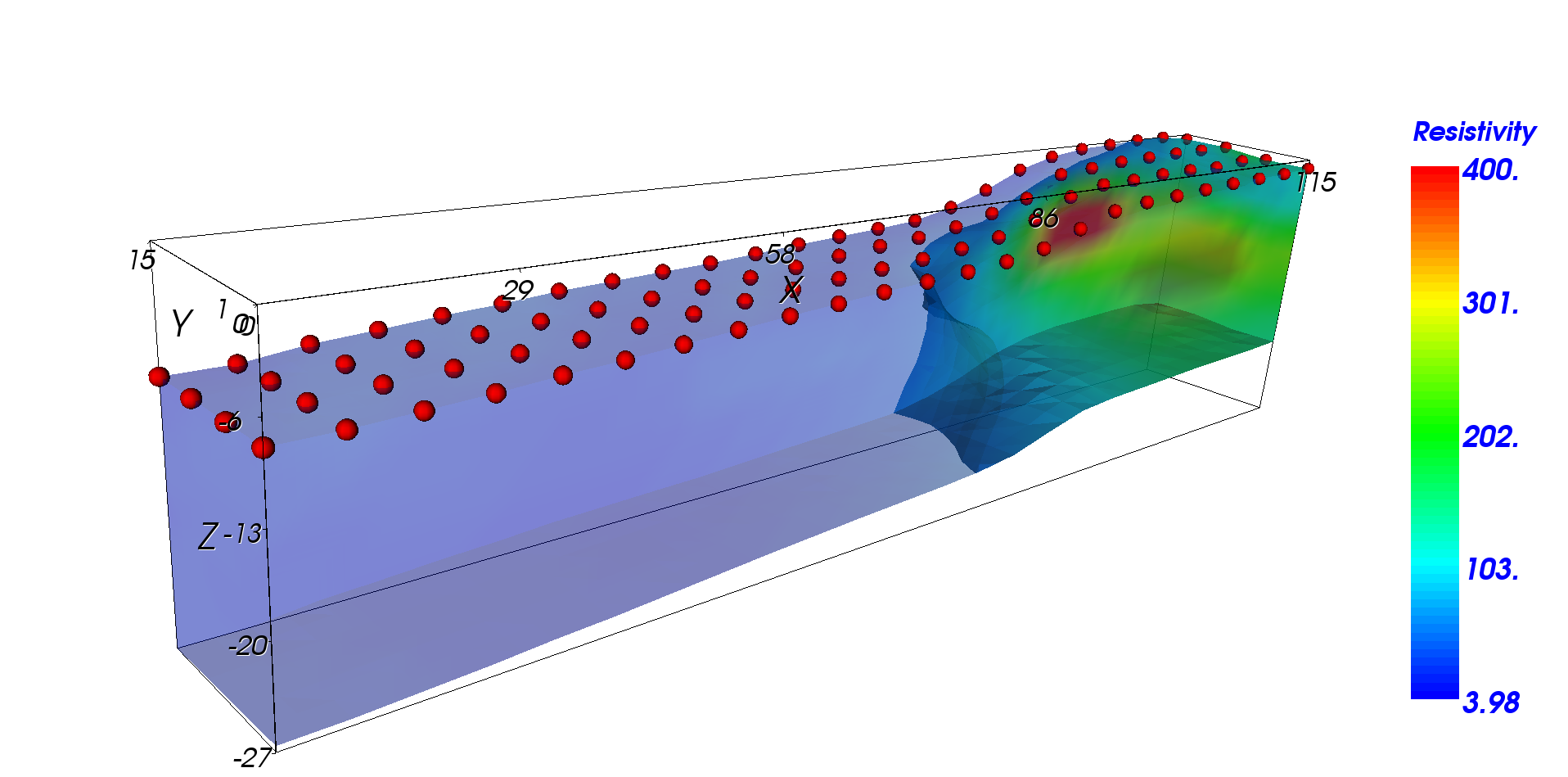

Supplement: Supplementary file 1 [file mmc1.zip › ERT Location 1/3D ERT/Ban-Huana-3D Topo3.ERTprj/Huana3.bmp]

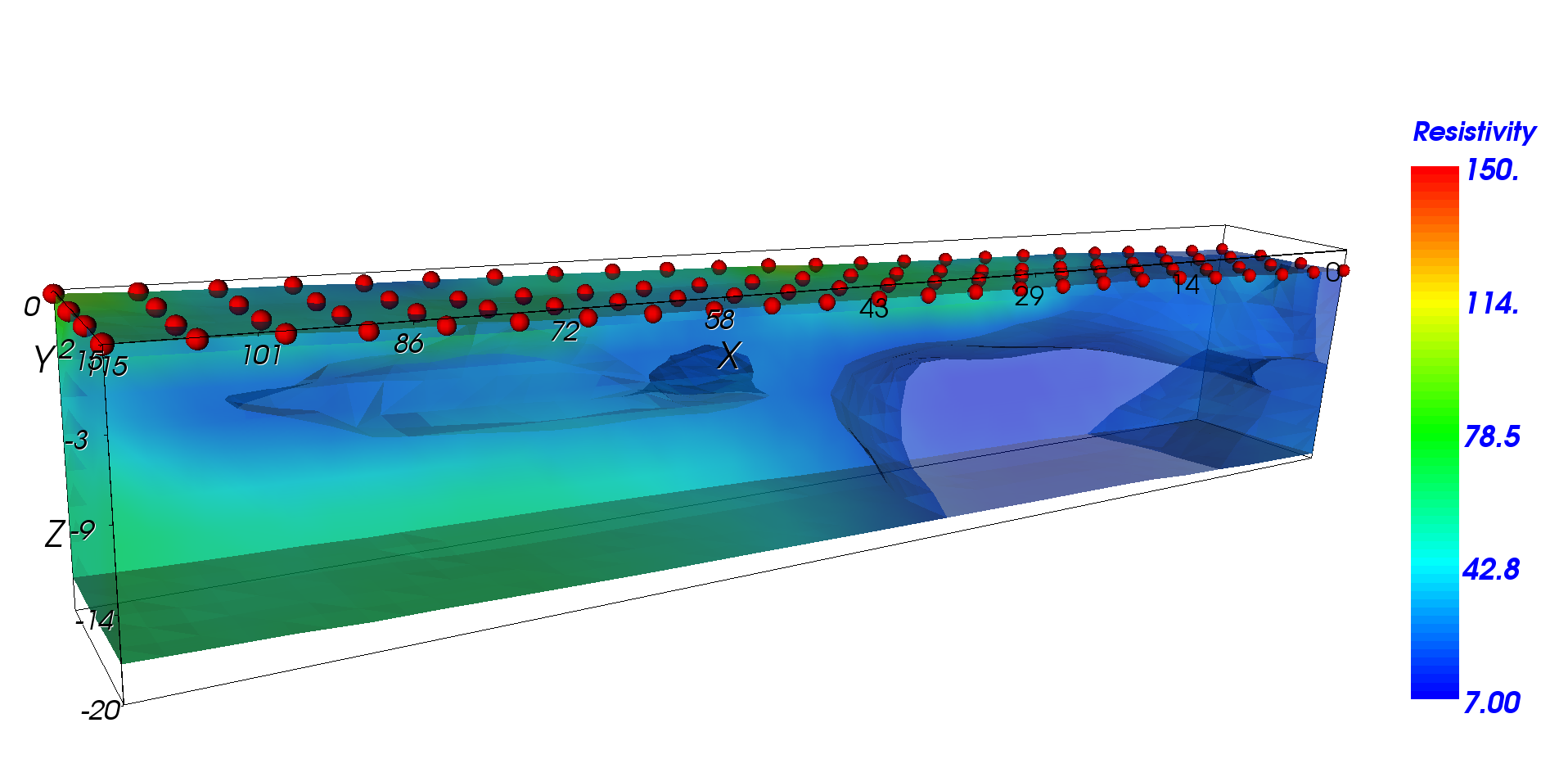

Supplement: Supplementary file 2 [file mmc2.zip › ERT Location 2/3D ERT/Ban-SamBon-3D Topo1.ERTprj/sambon.bmp]

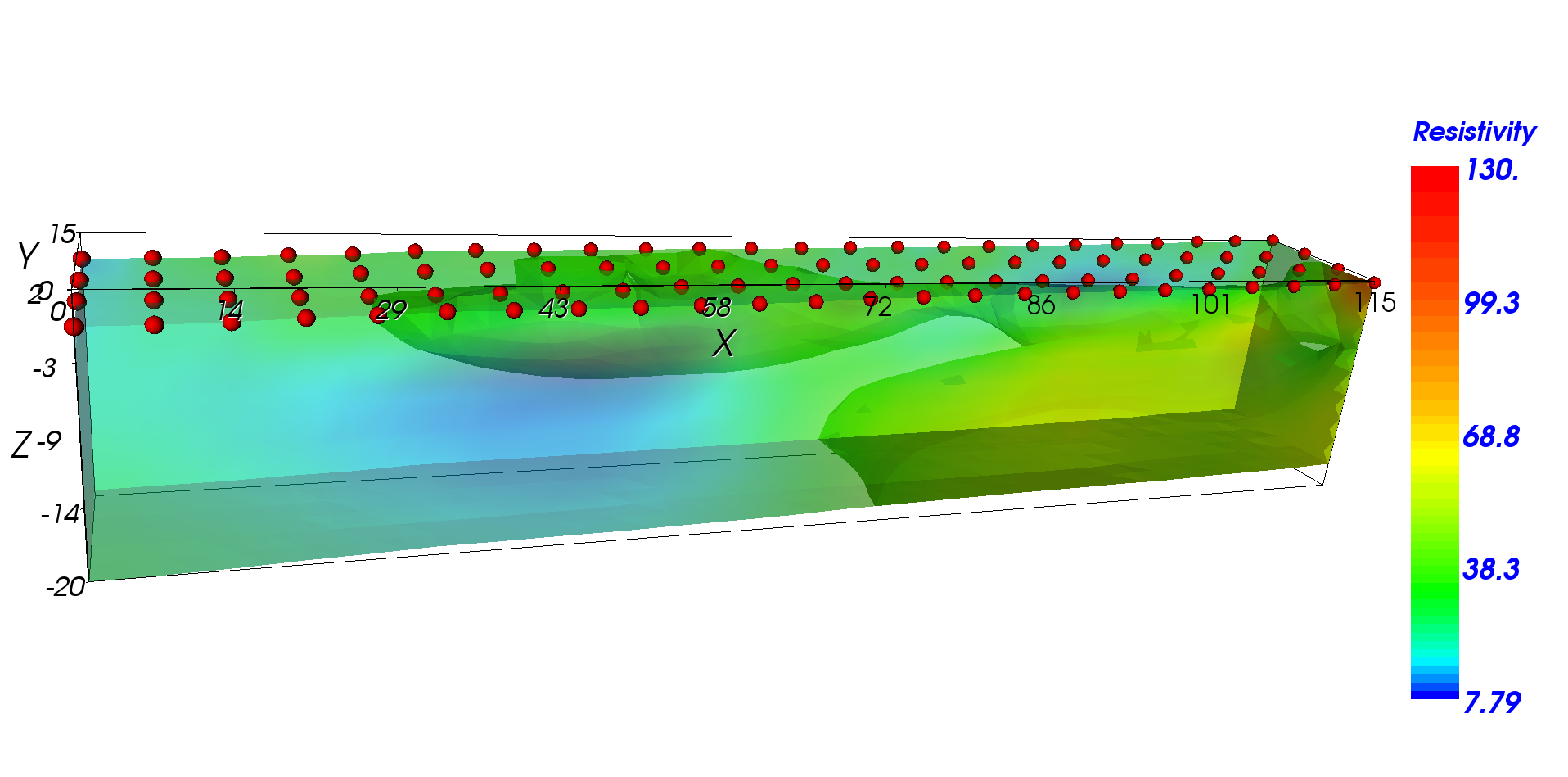

Supplement: Supplementary file 2 [file mmc2.zip › ERT Location 2/3D ERT/Ban-SamBon-3D Topo1.ERTprj/sambon2.bmp]

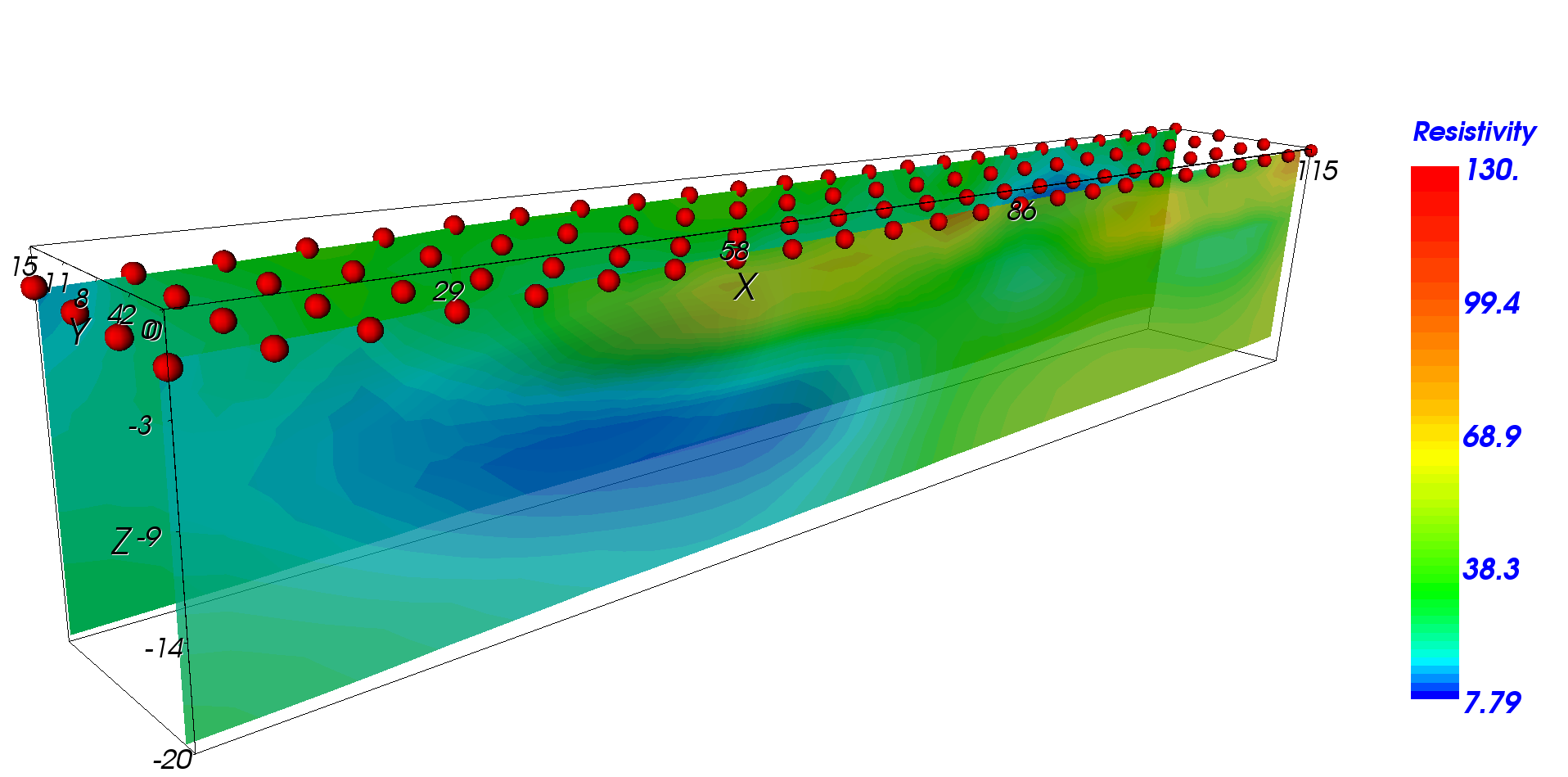

Supplement: Supplementary file 2 [file mmc2.zip › ERT Location 2/3D ERT/Ban-SamBon-3D Topo1.ERTprj/sambon3.bmp]

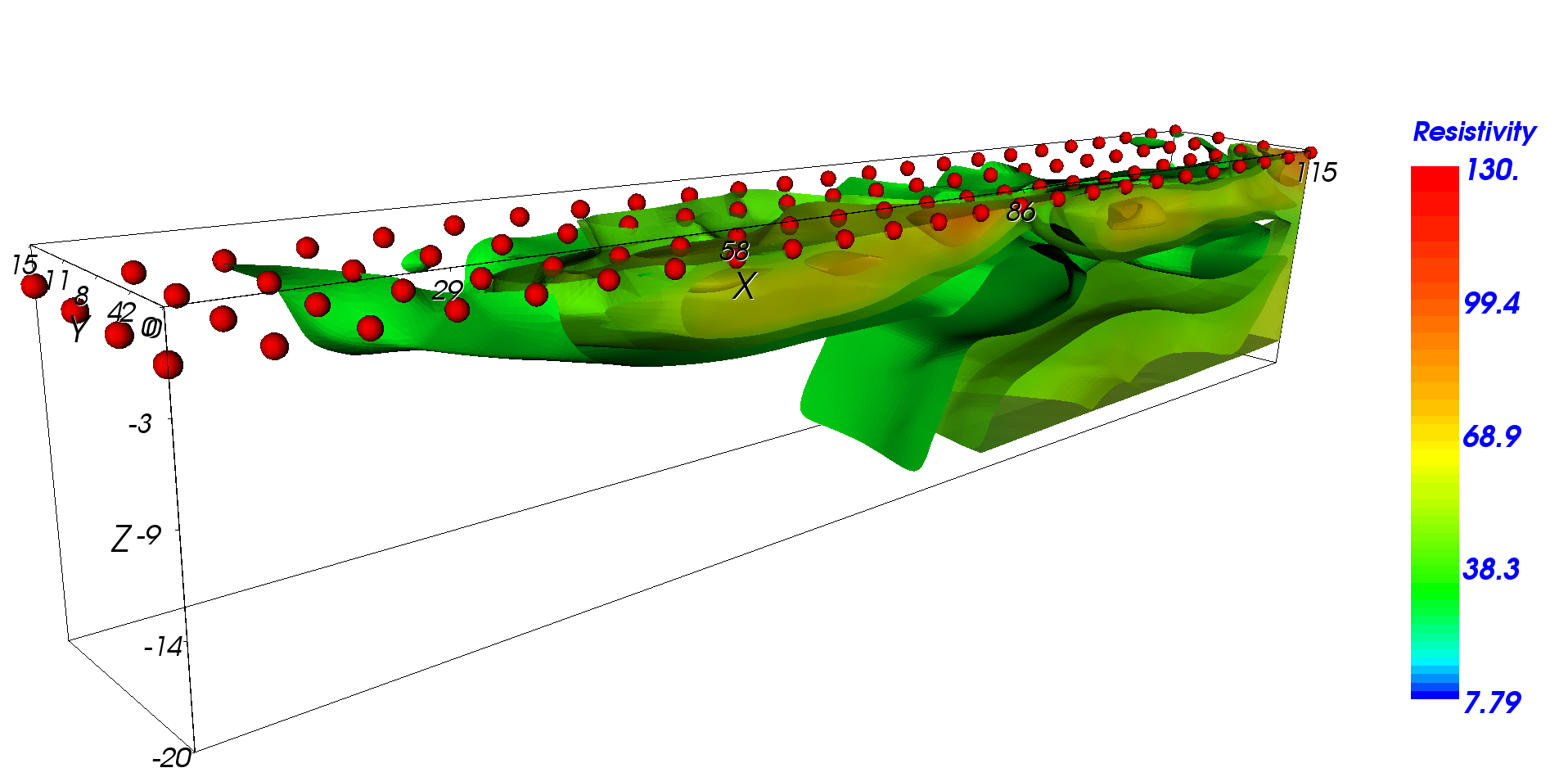

Supplement: Supplementary file 2 [file mmc2.zip › ERT Location 2/3D ERT/Ban-SamBon-3D Topo1.ERTprj/sambon4.bmp]
